# Oritavancin polymethylmethacrylate (PMMA)—compressive strength testing and in vitro elution

**DOI:** 10.1186/s13018-019-1080-6

**Published:** 2019-02-12

**Authors:** Suzannah M. Schmidt-Malan, Kerryl E. Greenwood-Quaintance, Lawrence J. Berglund, Jayawant Mandrekar, Robin Patel

**Affiliations:** 1 Division of Clinical Microbiology, Department of Laboratory Medicine and Pathology, Mayo Clinic, 200 First Street SW, Rochester, MN 55905 USA; 2 Division of Infectious Diseases, Department of Medicine, Mayo Clinic, Rochester, MN 55905 USA; 3 Division of Biomedical Statistics and Informatics, Department of Health Sciences Research, Mayo Clinic, Rochester, MN 55905 USA; 4 Materials Structural Testing Research Core, Mayo Clinic, Rochester, MN 55905 USA

**Keywords:** Oritavancin, Polymethylmethacrylate, Elution, Compressive strength

## Abstract

**Background:**

Polymethylmethacrylate (PMMA) is used for local antimicrobial delivery in orthopedic infection. Oritavancin is a long half-life lipoglycopeptide with broad activity against Gram-positive bacteria. Herein, we addressed if 7.5% *w*/*w* oritavancin mixed into PMMA affects PMMA strength and whether it elutes from PMMA, compared to vancomycin.

**Methods:**

Elution was assessed by placing an oritavancin- or vancomycin-loaded bead in a flow system with human plasma. Compressive strength of bland compared to oritavancin- or vancomycin-loaded PMMA was assessed after 0, 3, and 7 days of soaking in 1 ml of pooled normal human plasma at 37 °C, by testing to failure in axial compression using a servo-hydraulic testing machine.

**Results:**

Median compressive strength on days 0, 3, and 7 for bland PMMA compared to oritavancin- or vancomycin-loaded PMMA was 80.1, 79.4, and 72.4 MPa, respectively; 93.3, 86.4, and 65.3 MPa, respectively; and 97.8, 82.7, and 65.9 MPa, respectively. Oritavancin reduced PMMA compressive strength after 3 and 7 days (*P* = 0.0250 and 0.0039, respectively), whereas vancomycin reduced the PMMA compressive strength after 0, 3, and 7 days (*P* = 0.0039, 0.0039, and 0.0062, respectively) as compared to bland PMMA. Oritavancin-loaded PMMA had higher compressive strength than vancomycin-loaded PMMA on days 3 and 7 (*P* = 0.0039 and 0.0062, respectively). Compressive elastic moduli were 1226, 1299, and 1394 MPa for bland PMMA; 1253, 1078, and 1245 MPa for oritavancin-loaded PMMA; and 986, 879, and 779 MPa for vancomycin-loaded PMMA on days 0, 3 and 7, respectively. Oritavancin-loaded PMMA had higher compressive elastic moduli than vancomycin-loaded PMMA on days 0 and 7 (*P* = 0.0250 and 0.0062, respectively). Following polymerization, 1.0% and 51.9% of the initial amount of oritavancin and vancomycin were detected, respectively. *C*_max_, *T*_max_, and AUC_0–24_ were 1.7 μg/ml, 2 h, and 11.4 μg/ml for oritavancin and 21.4 μg/ml, 2 h, and 163.9 μg/ml for vancomycin, respectively.

**Conclusions:**

Oritavancin-loaded PMMA had higher compressive strength than vancomycin-loaded PMMA on days 3 and 7 and higher compressive elastic moduli than vancomycin-loaded PMMA on days 0 and 7. However, proportionally less oritavancin than vancomycin eluted out of PMMA.

## Introduction

Osteomyelitis and prosthetic joint infections (PJI) are inherently difficult to treat, with treatment typically including debridement, along with local and/or systemic antimicrobial therapy [1, 2].

For almost half a century, polymethylmethacrylate (PMMA) has been used for local antimicrobial delivery in the treatment and prevention of orthopedic infections [3], as it allows release of high concentrations of antibiotics at the site of infection [1, 2]. The specific antimicrobial(s) used, amount of antimicrobial(s) used, and porosity and type of cement affect release kinetics [4]. The glycopeptide vancomycin is widely used for this purpose because of its broad spectrum of activity, heat stability, and low allergic potential [5]. For example, vancomycin was employed in 13 of 18 patients with antimicrobial-loaded PMMA in a recent study from our institution [6]. Generally, 1 g (low dose) to 4 g (high dose) of vancomycin is added to 40 g of PMMA [6], with low dose being used for prophylaxis and high dose for treatment of established infection [1]. Notably, Gram-positive bacteria with reduced susceptibility to vancomycin have been identified [7, 8], and vancomycin has poor activity against Gram-positive bacteria in biofilms [9].

Oritavancin is a lipoglycopeptide active in vitro against resistant Gram-positive bacteria, including vancomycin-resistant enterococci, methicillin-resistant staphylococci, and penicillin-resistant streptococci [10–12]. Oritavancin inhibits transglycosylation and transpeptidation of peptidoglycan and disrupts the integrity of the bacterial cell membrane [8]. It has a long elimination half-life [13], a property that may make it useful for incorporation in PMMA for local antimicrobial delivery. The addition of antimicrobials to PMMA has the potential to weaken the strength of PMMA [14], and as mentioned, release from PMMA is not uniform for all antimicrobial agents. Here, we tested the strength of PMMA with the addition of oritavancin and evaluated elution of oritavancin from PMMA, using vancomycin as a comparator to determine if this antimicrobial may be useful in orthopedic procedures involving antimicrobial agent-loaded PMMA.

## Materials and methods

### PMMA preparation

PMMA (Simplex P, Stryker©, Kalamazoo, MI) was mixed per the manufacturer’s guidelines. 7.5% *w*/*w* oritavancin or vancomycin was added to the PMMA which was formed into 3-mm beads (for elution studies) or 6 mm × 12 mm cylinders (for strength testing studies), and allowed to polymerize for 24 h [15, 16]. Beads and cylinders were stored at 4 °C and weighed prior to use. Beads were prepared three ways to determine the best method for prevention of oritavancin binding to the mold surface: (1) PMMA with oritavancin, (2) PMMA with oritavancin plus 0.002% polysorbate 80, and (3) oritavancin in 0.002% polysorbate 80 pre-coated mold with PMMA with oritavancin. For the last approach, the mold was pre-coated with a 5 mg/ml oritavancin solution in 0.002% polysorbate 80, by pipetting the solution over the mold and then rinsing it with sterile deionized water, followed by air drying for several minutes. A positive control consisting of 7.5% oritavancin in pooled normal human plasma (PNHP) was tested. A bland PMMA bead was prepared and assayed as above as a negative control. The effect of PMMA components and polymerization on antimicrobial activity was determined by homogenizing a bead and placing it in 1 ml of PNHP. The pre-coated mold was determined to be the ideal strategy and was used for all subsequent preparation of beads and cylinders.

### Mechanical strength testing

Compressive strength of bland compared to oritavancin- or vancomycin-loaded PMMA was assessed after 0, 3, and 7 days of soaking in 1 ml of PNHP at 37 °C. ISO standard 5883 for bone cement, which specifies physical and mechanical requirements for cement used in the internal fixation of orthopedic prostheses, was followed [17]. Six cylindrical samples for each time point were tested to failure in axial compression using a servo-hydraulic testing machine (MTS Systems Corporation model 810, Eden Prairie, MN). The rate of testing was 20 mm/min. Data of force and displacement were converted to stress and strain and analyzed for 2% offset compressive strength and compressive elastic modulus.

### Antimicrobial elution

**Fig. 1:**
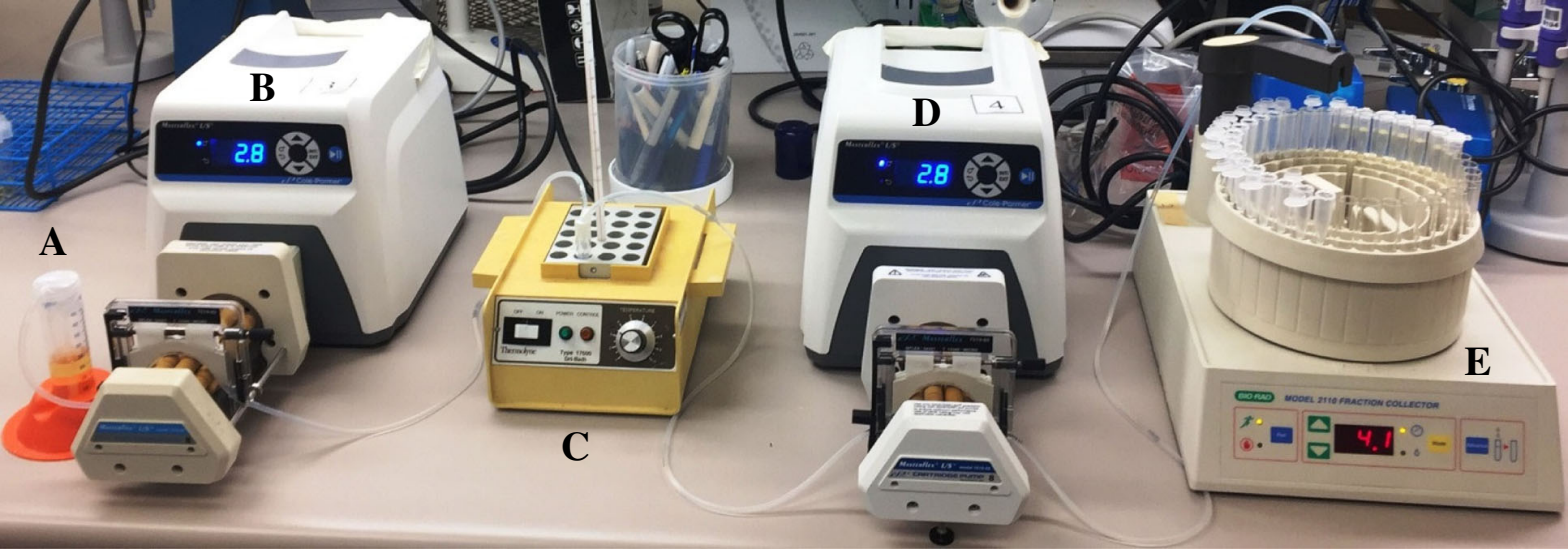
Flow system used to collect hourly samples. **a** Stock of sterile PNHP. **b** Masterflex L/S Precision Pump (Cole Parmer, Vernon Hills, IL). **c** Heat block (Barnstead/Thermolyne, Model 0817615, Dubuque, IA) holding tube containing PMMA bead. **d** Masterflex L/S Precision Pump. **e** Fraction collector (BioRad, Model 2110, Richmond, CA)

Antimicrobial elution was determined by placing a bead in 1 ml of PNHP, at 37 °C, in a continuous flow chamber with 1 ml/h PNHP flow, with the effluent collected hourly for 24 h, using a modified previously described flow system (Fig. 1) [15]. Oritavancin concentrations were determined by high-performance liquid chromatography (HPLC) and vancomycin concentrations by bioassay. For the vancomycin bioassay, 20 μl of each sample was added to 6-mm sterile blank paper discs (Becton, Dickinson and Company, Sparks, MD) and placed on Mueller Hinton agar plates containing *Bacillus subtilis* ATCC 6633. The zones of inhibition were measured. These were compared with standard curves to determine the antimicrobial concentrations. Standard curves were made by serial dilutions in PNHP to make select reference concentrations ranging from 4 to 12 μg/ml. The collected effluent for the 4- and 5-h samples were diluted 1:3 and 1:2, respectively, before the assay to assure that the antimicrobial concentration was in the range of the standards. Maximum antimicrobial concentrations, the time at which maximum concentration was reached, area under the concentration-time curve, and percent antibiotic recovered from the beads were reported as means. Testing was performed in triplicate.

### Statistical methods

Descriptive summaries for the strength testing were reported as median (minimum, maximum) for compressive strength as a continuous variable. Compressive strength and compressive elastic modulus for PMMA cylinders among the three groups (bland, with oritavancin or vancomycin) were compared using the Kruskal Wallis test. If the overall differences among the groups were significant, further pairwise comparisons were made using Wilcoxon rank-sum tests. Similar analyses were performed when comparing compressive strengths across days (i.e., 0, 3, 7 days) within each of the three groups. All statistical tests were two-sided with an alpha level of 0.05. Analysis was performed using SAS version 9.4 (SAS Inc., Cary, NC). Due to small sample sizes, no adjustments were made for multiple comparisons.

## Results

### PMMA preparation

The ideal method for making the beads involved pre-coating the mold with a concentrated solution of oritavancin in 0.002% polysorbate 80, before placing the PMMA with oritavancin into the mold. Using this method, the mean concentration (fraction of total amount of drug incorporated) of oritavancin after homogenization was 7.0 standard deviation (SD) ± 0.17 μg/ml (1.0%) (Table 1). The mean concentration of vancomycin after homogenization was 865.4 SD ± 238.7 μg/ml (51.9%).

**Table 1:** Concentrations of oritavancin eluted from 7.5% *w*/*w* oritavancin polymethylmethacrylate (PMMA) beads prepared using three methods

Sample	Mean (*n* = 3) measured concentration (μg/ml)	Standard deviation (*n* = 3) (μg/ml)
PMMA alone (negative control)	Below quantifiable limit	Not applicable
PMMA + oritavancin	2.7	0.07
PMMA + oritavancin + polysorbate 80	3.0	0.08
Oritavancin pre-coated mold^a^	7.0	0.17
Oritavancin in plasma, no PMMA (positive control)	62.3	1.2

Oritavancin levels were assessed following homogenization of beads and mixing homogenate with pooled normal human plasma

^a^PMMA + oritavancin + polysorbate 80

### Mechanical strength testing

**Fig. 2:**
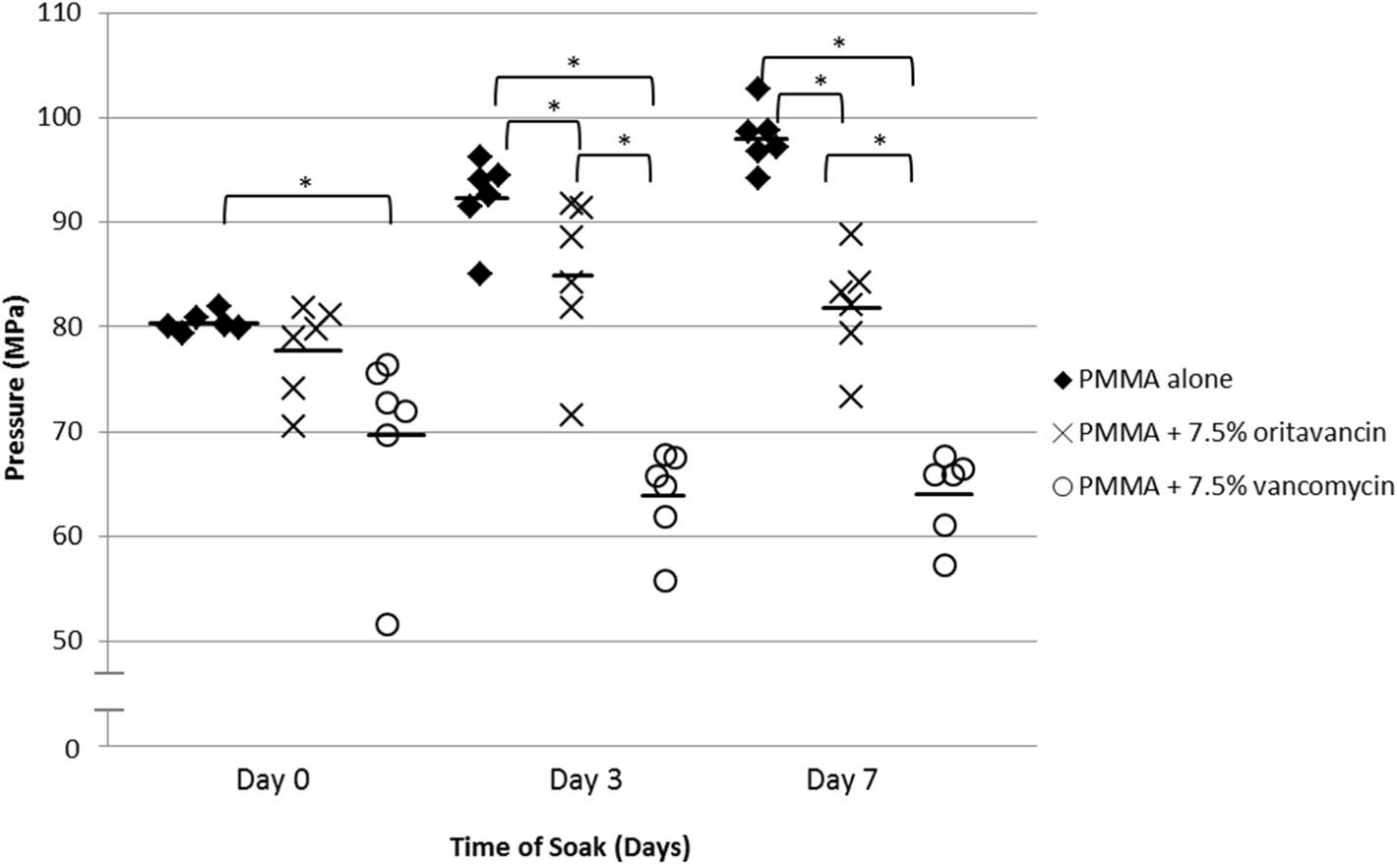
Compressive strength of cylinders tested after soaking in pooled normal human plasma for 0, 3, and 7 days. *P < 0.05; *n* = 6; solid black lines represent the mean value of each group; PMMA, polymethylmethacrylate

The median (minimum, maximum) 2% offset compressive strengths for PMMA alone on days 0, 3, and 7 were 80.1 (79.4, 81.9), 93.3 (85.1, 96.3), and 97.8 (94.2, 102.7) MPa, respectively. For PMMA with oritavancin, the median (minimum, maximum) 2% offset compressive strengths on days 0, 3, and 7 were 79.4 (70.5, 81.8), 86.4 (71.6, 91.8), and 82.7 (73.3, 88.8) MPa, respectively. The median (minimum, maximum) 2% offset compressive strengths for PMMA with vancomycin on days 0, 3, and 7 were 72.4 (51.6, 76.5), 65.3 (55.8, 67.8), and 65.9 (57.3, 66.5) MPa, respectively (Fig. 2). When comparing bland PMMA to PMMA with oritavancin on day 0, there was no difference in strength (*P* = 0.2623); however, bland PMMA was stronger than PMMA with vancomycin (*P* = 0.0039). PMMA with oritavancin remained above the ISO requirement of ≥ 70 MPa over the 7 days of testing, whereas this was not the case with PMMA with vancomycin on days 3 and 7 [14]. On day 3, bland PMMA was stronger than either PMMA with oritavancin or vancomycin (*P* = 0.0250 and 0.0039, respectively), and PMMA with oritavancin was stronger than PMMA with vancomycin (*P* = 0.0039). On day 7, bland PMMA was stronger than PMMA with oritavancin or vancomycin (*P* = 0.0039 and 0.0062, respectively), and PMMA with oritavancin was stronger than PMMA with vancomycin (*P* = 0.0062).

**Fig. 3:**
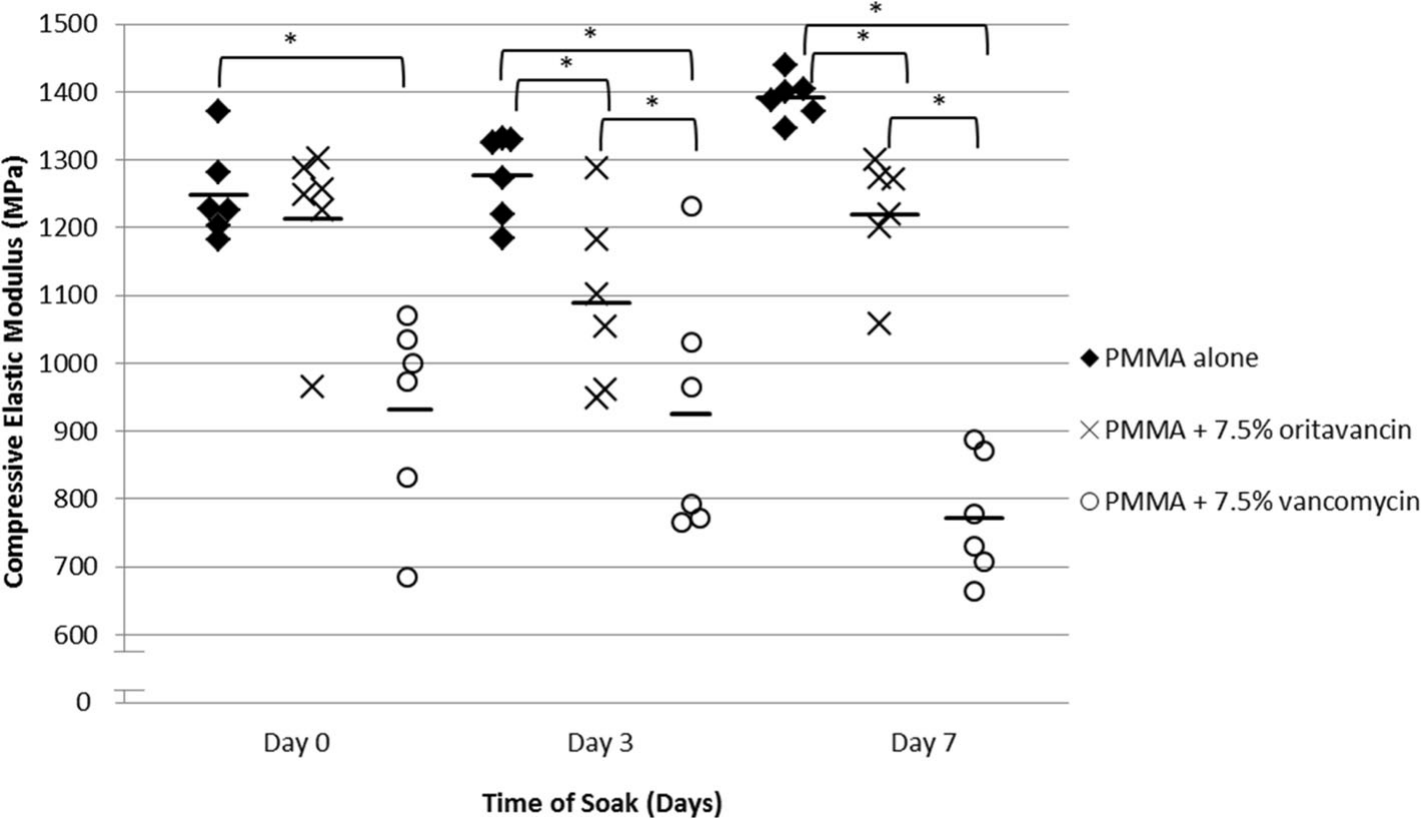
Compressive elastic modulus of cylinders tested after soaking in pooled normal human plasma for 0, 3, and 7 days. *P < 0.05; *n* = 6; solid black lines represent the mean value of each group; PMMA, polymethylmethacrylate

The compressive elastic modulus of bland PMMA on days 0, 3, and 7 was 1226, 1299, and 1394 MPa, respectively. The compressive elastic modulus of PMMA with oritavancin on days 0, 3, and 7 was 1253, 1078, and 1245 MPa, respectively. The compressive elastic modulus of PMMA with vancomycin on days 0, 3, and 7 was 986, 879, and 779 MPa, respectively (Fig. 3). On day 0, the compressive moduli were not significantly different between bland PMMA and PMMA with oritavancin (*P* = 0.6310), but were significantly different between bland PMMA and PMMA with vancomycin (*P* = 0.0039) and PMMA with oritavancin compared to PMMA with vancomycin (0.0250). On day 3, the compressive elastic modulus for bland PMMA was significantly different compared to PMMA with oritavancin or vancomycin (*P* = 0.0163 and 0.0104, respectively), but not between PMMA with oritavancin and vancomycin (*P* = 0.1495). On day 7, the compressive elastic modulus for bland PMMA was significantly different compared to PMMA with oritavancin or vancomycin (*P* = 0.0039 and 0.0062, respectively) and was also significantly different between PMMA with oritavancin and PMMA with vancomycin (*P* = 0.0062).

### Antimicrobial elution

**Fig. 4:**
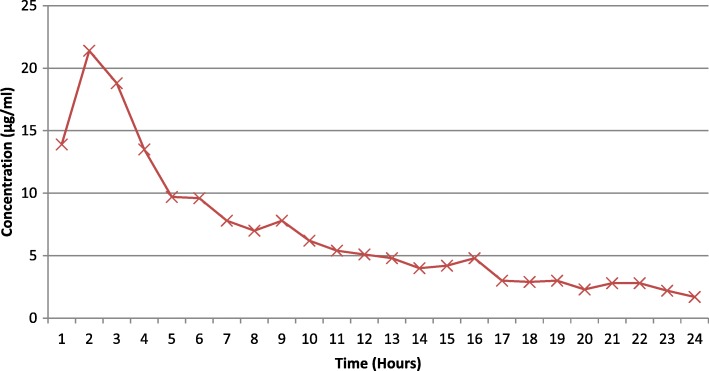
Vancomycin bead elution over 24 h (*n* = 3). Average concentration of the eluate of vancomycin beads over 24 h at 37 °C; *n* = 3

**Fig. 5:**
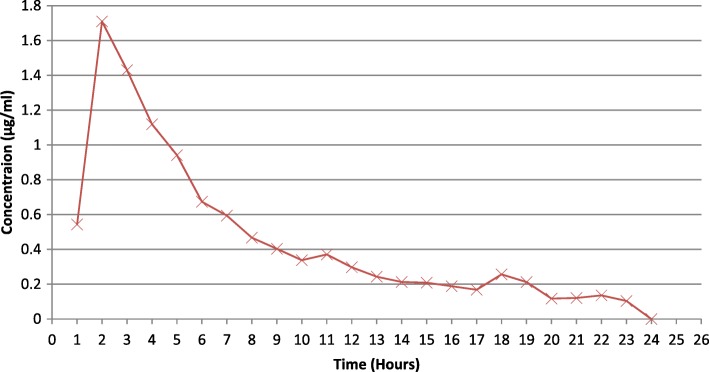
Oritavancin bead elution over 24 h (*n* = 3). Average concentration of the eluate of oritavancin beads over 24 h at 37 °C; *n* = 3

Elution profiles for oritavancin and vancomycin are presented in Table 2. The *C*_max_, *T*_max_, and AUC_0–24_ were 1.7 μg/ml, 2 h, and 11.4 μg/ml for oritavancin and 21.4 μg/ml, 2 h, and 163.9 μg/ml for vancomycin, respectively. The mean 24h cumulative percent elution of oritavancin was 1.6% compared to 9.4% for vancomycin. The antimicrobial concentrations at each time point are shown in Figs. 4 and 5.

**Table 2:** Elution profiles for oritavancin (*n* = 3) and vancomycin (*n* = 3) from polymethylmethacrylate (PMMA)

	Mean *C*_max_ (μg/ml)	Mean *T*_max_ (h)	Mean AUC_0–24_ (μg/ml/h)	Mean % elution
Oritavancin	1.7 ± 1.7	2	11.4	1.6%
Vancomycin	21.4 ± 4.0	2	163.9	9.4%

*C*_*max*_ maximum concentration, *T*_*max*_ time at which the maximum concentration was reached, *AUC*_*0–24*_ area under the concentration-time curve from 0 to 24 h

## Discussion

PMMA with oritavancin was significantly stronger than PMMA with vancomycin as shown by results of compressive strength testing on days 3 and 7 and compressive elastic modulus evaluation on days 0 and 7. Lee et al. evaluated strength and elution of several different brands of PMMA (Simplex P, Osteobond, PALACOS R, and Depuy-CMW) with several different brands of vancomycin (Vanco, Lyo-Vancin, and sterile vancomycin from Hospira Inc.) and showed that regardless of the brand of PMMA, compression strength of high doses (4 g/40 g of PMMA powder) of sterile vancomycin after 14 days of elution did not exceed 70 MPa, the specified minimum strength requirement of PMMA according to ISO 5833 (E) [1, 17]. In our study, with 3 g of vancomycin, we also observed that the compressive strength of PMMA did not exceed 70 MPa on days 3 and 7. It is possible that as vancomycin, a large molecule, elutes from PMMA, it creates voids in PMMA lowering the mechanical strength [1]. Although the compressive strength of PMMA alone was more than that of PMMA with oritavancin after 3 and 7 days in PNHP, the strength of PMMA with oritavancin exceeded the minimum strength requirement of ≥ 70 MPa at all times tested.

Oritavancin non-specifically binds many surfaces, including borosilicate glass, polypropylene, polystyrene, polymethyl pentene, and Teflon [18]. This binding is saturable and can be overcome by pre-treating surfaces with a concentrated solution of oritavancin. We evaluated different strategies to prepare PMMA with oritavancin to overcome non-specific binding—placing PMMA and oritavancin powder directly into the mold, adding 0.002% polysorbate 80 to PMMA and oritavancin, and finally pre-coating the mold with a concentrated solution of oritavancin before adding PMMA with oritavancin. When making the beads with just PMMA and oritavancin powder without pre-coating the mold, the beads were visibly hollowed, possibly as a result of oritavancin interactions with the mold surface. Overall, pre-coating with a high concentration of oritavancin and use of polysorbate 80 alongside oritavancin was the ideal strategy evaluated. Because PMMA setting is exothermic, the setting process might influence the antibiotic potency of oritavancin; however, as oritavancin is a vancomycin derivative, and vancomycin was not as affected, this is unlikely to be the case [1, 5, 19]. Therefore, the low levels are likely due to non-specific binding and inability to release from PMMA.

Under our experimental conditions, less oritavancin is eluted from PMMA than vancomycin. This is also the case when comparing the elution of oritavancin in the present study to the elution of tobramycin, amikacin, gentamicin, daptomycin, cefazolin, ciprofloxacin, gatifloxacin, levofloxacin, linezolid, and rifampin in prior studies [15, 16, 20, 21]. As mentioned, the low level of elution of oritavancin may be because it readily binds to surfaces, possibly including PMMA itself, rendering it unavailable for release [18]. In a review by Cui et al., it was found that as compared with commercially mixed antibiotic-loaded bone cement, hand-mixed antibiotic bone cement did not result in homogenous dispersion in the bone cement, which can decrease elution [4]; theoretically, this may have affected our results, as our PMMA was hand-mixed. However, in our prior studies using hand-mixed PMMA, we did not witness a low level of elution using other antibiotics [15, 20–22].

By measures of MIC_90_, oritavancin is approximately four- to eightfold more potent than vancomycin against *Staphylococcus aureus* and vancomycin-susceptible enterococci [11, 12]. However, despite this greater comparative in vitro potency, the low-level elution of oritavancin from PMMA may limit the utility of oritavancin-PMMA for local antimicrobial delivery. Strategies to improve its elution, such as the addition of porogens (e.g., dextran, glycine, xylitol, gelatin sponge, ceramic granules), to increase PMMA porosity and surface area should be explored [19, 23–26].

## Conclusions

Overall, although the addition of oritavancin did not reduce the strength of PMMA as much as did vancomycin, its relatively low level of elution may limit its value for local delivery through PMMA.

## Abbreviations

AUC_0–24_: Area under the concentration-time curve from 0 to 24 h
*C*_max_: Maximum concentration
HPLC: High-performance liquid chromatography
PJI: Prosthetic joint infection
PMMA: Polymethylmethacrylate
PNHP: Pooled normal human plasma
*T*_max_: Time at which the maximum concentration was reached
